# Monocyte Chemotactic Protein-1 (MCP-1) and Growth Factors Called into Question as Markers of Prolonged Psychosocial Stress

**DOI:** 10.1371/journal.pone.0007659

**Published:** 2009-11-03

**Authors:** Ingibjörg H. Jonsdottir, Daniel A. Hägg, Kristina Glise, Rolf Ekman

**Affiliations:** 1 The Institute of Stress Medicine, Gothenburg, Sweden; 2 Institute of Neuroscience and Physiology, Sahlgrenska Academy, University of Gothenburg, Gothenburg, Sweden; Agency for Science, Technology and Research (A*STAR), Singapore

## Abstract

**Background:**

Psychosocial stress is becoming a major contributor to increased mental ill-health and sick leave in many countries. Valid markers of chronic stress would be valuable for diagnostic and prognostic purposes. A recent study suggested monocyte chemotactic protein-1 (MCP-1), epidermal growth factor (EGF) and vascular endothelial growth factor (VEGF) as markers of chronic stress. We aimed to confirm these potential biomarkers of prolonged psychosocial stress in female patients.

**Methodology/Principal Findings:**

Circulating levels of MCP-1, EGF and VEGF, along with several other cytokines, were measured in plasma from 42 female patients suffering from exhaustion due to prolonged psychosocial stress and 42 control subjects, using a protein biochip immunoassay. There were no significant differences between patients and controls in any of the cytokines or growth factors analyzed. Furthermore, when using a different protein bioassay and reanalyzing MCP-1 and VEGF in the same samples, markedly different levels were obtained. To further explore if inflammation is present in patients with exhaustion, the classical inflammatory marker C-reactive protein (CRP) was measured in another group of patients (n = 89) and controls (n = 88) showing a small but significant increase of CRP levels in the patients.

**Conclusions/Significance:**

MCP-1, EGF and VEGF may not be suitable markers of prolonged psychosocial stress as previously suggested. Furthermore, significant differences were obtained when using two different protein assays measuring the same samples, indicating that comparing studies where different analytic techniques have been used might be difficult. Increased levels of CRP indicate that low-grade inflammation might be present in patients with exhaustion due to prolonged stress exposure but this inflammation does not seem to be reflected by increase in circulating MCP-1 or other cytokines measured.

## Introduction

Increased long-term sick leave is a substantial problem in many countries, and a major reason for this increase is generally believed to be related to psychosocial stress [1], [2], [3]. This increase is more pronounced among women, and factors including high demands, increased work load, changes in the organisation and psychological harassment are believed to contribute to the increased stress-related mental health problems in the working population [1]. Burnout is one of several mental health variables defined as a psychological outcome of long term stress, and emotional exhaustion is thought to be an important manifestation of burnout [4], [5]. Vital exhaustion is another term often used to define the mental health consequences of prolonged psychosocial stress [6]. Work-related stress is also associated with common mental ill-health such as depression and anxiety [7].

The biological consequences of chronic stress exposure leading to mental ill-health such as burnout or exhaustion are still not well known. Many studies focus on the hypothalamus-pituitary-adrenal (HPA) axis and different cortisol measurements [8] and the question remains how other biological systems might be affected. One interesting aspect of biological consequences of prolonged stress is low-grade inflammation. Inflammatory markers such as C-reactive protein (CRP) and interleukin-6 are associated with depression [9], and inflammatory processes have been suggested to be an important link between psychosocial stress and cardiovascular diseases such as atherosclerosis [10].

Asberg and co-workers recently showed that monocyte chemotactic protein-1 (MCP-1) is elevated in female patients with stress-related mental disorders when compared to healthy controls [11]. MCP-1 is a chemokine with important immune functions, such as the recruitment of monocytes into the arterial wall, promoting atherosclerosis [12]. Epidermal growth factor (EGF) and vascular endothelial growth factor (VEGF) were also shown to be elevated in the patients studied by Asberg and co-workers [11]. This promising data raises the question if these markers can be used as biochemical markers of psychosocial stress and the authors called for more research to confirm their findings. Therefore, the present study was designed as an attempt to confirm if the suggested markers (MCP-1, EGF and VEGF) are elevated in patients with stress-related exhaustion. We also measured the more common marker of inflammation CRP in a different group of patients with stress-related exhaustion.

## Materials and Methods

### Subjects and Diagnostic Procedures

All patients included in this study where referred to an outpatient clinic at the Institute of Stress Medicine (ISM) located in Gothenburg, Sweden. The patients were ambulatory at the time of the study and none had received inpatient care due to their illness. They were referred from primary care units or occupational health care centres from the western part of Sweden and the referral criteria was 1) “probable Exhaustion Disorder” with no apparent somatic disorder or abuse that could explain the exhaustion and 2) a maximal duration of sick leave of six months. Healthy control subjects were recruited from an ongoing longitudinal cohort study of the Västra Götaland Region (mainly health care workers and social insurance officers) and from advertisements in daily newspapers.

The first part of the study (cytokines and growth hormone measurements) involves female patients with stress-related exhaustion (n = 42) and healthy female controls (n = 42). Of the patients, 52% were on full time sick-leave and 21% where on part-time sick-leave.

A senior physician at the clinic carried out a diagnostic procedure of the patients, obtaining an extended anamnesis with a clinical examination. The DSM-IV based instrument PRIME-MD (one page patient questionnaire) was filled in by the patient and the results were used as a support in the diagnostic procedure. Whenever necessary this included a structured interview to identify any presence of mood and/or anxiety disorders. To obtain a relatively homogenous population of patients regarding stress-related exhaustion the diagnostic criteria for exhaustion disorder (ED) was used as inclusion criteria (Table 1). Thus all patients fulfilled the ED criteria, 74% also fulfilled the diagnostic criteria for depression and 64% for an anxiety disorder. Characteristics of the participants included in this part are shown in Table 2.

**Table 1:** Diagnostic criteria for Exhaustion Disorder according to The National Insurance Board in Sweden.

**A**	Physical and mental symptoms of exhaustion with minimum two weeks duration. The symptoms have developed in response to one or more identifiable stressors which have been present for at least 6 months.
**B**	Markedly reduced mental energy, which is manifested by reduced initiative, lack of endurance, or increase of time needed for recovery after mental efforts.
**C**	At least four of the following symptoms have been present most of the day, nearly every day, during the same 2-week period:
*1*	*Persistent complaints of impaired memory.*
*2*	*Markedly reduced capacity to tolerate demands or to work under time pressure.*
*3*	*Emotional instability or irritability.*
*4*	*Insomnia or hypersomnia.*
*5*	*Persistent complaints of physical weakness or fatigue.*
*6*	*Physical symptoms such as muscular pain, chest pain, palpitations, gastrointestinal problems, vertigo or increased sensitivity to sounds.*
**D**	The symptoms cause clinically significant distress or impairment in social, occupational or other important areas of functioning.
**E**	The symptoms are not due to the direct physiological effects of a substance (e.g. a drug of abuse, a medication) or a general medical condition (e.g. hypothyroidism, diabetes, infectious disease).
**F**	If criteria for major depressive disorder, dysthymic disorder or generalized anxiety disorder are met, exhaustion disorder is set a co-morbid condition.

**Table 2:** Descriptive data of patients and healthy controls, all females, included in the first part of the study (cytokine and growth factors measurements).

	Patients (n = 42)	Controls (n = 42)	p-value
**Age (years)** ¤	42.1±9.4	42.7±7.5	0.664
**BMI (kg/m^2^)** ¤	23.1±3.0	23.4±2.6	0.371
**Burnout score** ¤	5.4±0.81	2.6±1.22	<0.001
- Percent scoring >4.3 on burnout	88%	13%	
**Percent with depression**	74%	0%	
**(clinical diagnoses)**			
**Percent with anxiety**	64%	0%	
**(clinical diagnoses)**			
**HAD Depression Score** #			<0.001
0–6	36%	91%	
7–10	36%	2%	
>10	29%	7%	
**HAD Anxiety Score** #			<0.001
0–6	5%	74%	
7–10	21%	14%	
>10	74%	12%	

¤ Mann-Whitney test, data presented as mean and SD.

# Chi-square test, data presented as percentage within each scoring group.

The exclusion criteria for both patients and healthy controls were known systematic or psychiatric disease (except depression, anxiety and exhaustion for the patients), present infection, pregnancy, breast-feeding, oral contraceptives containing oestrogen, any medication with systemic effects (except for antidepressants for the patients), body mass index below 18.5 or over 30 kg/m^2^, vitamin B_12_ deficiency, thyroid disorders or over-consumption of alcohol. Thirty-one percent (n = 13) of the patients were using antidepressant medication and ten patients and four controls reported use of nicotine.

### Subjects (CRP Measurements)

In a second part of the study, CRP was measured in serum from 89 patients and 88 healthy controls, recruited and diagnosed in the same manner as described above using the same inclusion/exclusion criteria, thus all patients shared the feature of fulfilling the clinical criteria for ED. In this second group, 73% of the patients also fulfilled the diagnostic criteria for depression and 75% for anxiety disorder. The characteristics of the patients and healthy controls included in this part of the study are shown in Table 3. Thirty-three percent (n = 29) of the patients where on antidepressants when blood samples were taken.

**Table 3:** Descriptive data for subjects included in the second part of the study (CRP measurement).

	Patients (n = 89)	Controls (n = 88)	p-value
**Age (years)** ¤	40.9±6.7	44.6±8.5	0.006
**Sex (% male)** #	48%	50%	0.822
**BMI (kg/m^2^)** ¤	24.3±3.0	23.7±2.4	0.198
**Burnout score** ¤	5.3±0.85	2.3±0.88	<0.001
- Percent scoring >4.3 on burnout	92%	6%	
**Percent with depression**	73%	0%	
**(clinical diagnoses)**			
**Percent with anxiety**	75%	0%	
**(clinical diagnoses)**			
**HAD Depression Score** #			<0.001
0–6	34%	94%	
7–10	33%	6%	
>10	32%	0%	
**HAD Anxiety Score** #			<0.001
0–6	11%	83%	
7–10	24%	11%	
>10	64%	6%	

¤ Mann-Whitney test, data presented as mean and SD.

# Chi-square test, data presented as percentage within each scoring group.

### Ethics Statement

The study was approved by The Regional Ethical Review Board in Gothenburg, Sweden and was conducted according to the Helsinki Declaration. All subjects included in the study gave written informed consent.

### Exhaustion Disorder Criteria

According to the International Classification of Diseases (ICD-10), burnout is categorized as “Problems related to life-management difficulty”, but other than that, burnout syndrome is not a clinical defined illness. In order for physicians to better define people suffering from stress-related disease with pronounced mental and physical exhaustion, The National Insurance Board in Sweden recently proposed a clinical diagnostic criteria that could be used in clinical practice, referred to as “Exhaustion Disorder” (ED). The ICD-10 code F43.8 is used for this diagnose in clinical practice. To be diagnosed with ED it is required that the physician together with the patient is able to identify one or more stressors that have been present for at least six months, during which time the symptoms have developed. The criteria do not specify the type or intensity of the stress exposure but it is implicit that it should be significant enough to provoke the stress symptoms. The physician goes through the complete ED diagnostic criteria (Table 1) with the patient. A and B are obligatory criteria as well as the presence of at least four of six symptoms listed under C. Also, the condition should cause significant distress and/or impairment of important areas of functioning (D) and no symptoms should be due to direct physiological effects of a substance or a general medical condition (E). Finally, if the patient does meet the criteria for major depressive disorder, dysthymic disorder or generalised anxiety disorder, these diagnoses are set first and ED is set as a co-morbid condition. In this study special attention is held to chronic fatigue syndrome, and fibromyalgia sharing some common symptoms with ED and those patients where excluded from this study.

### Self-Rated Anxiety and Depression

The Hospital Anxiety and Depression (HAD) scale was used to assess self-reported depression and anxiety in both patients and controls. It was originally developed for non-psychiatric clinics to detect states of depression and anxiety [13] and the scale has been found to perform well in assessing cases of anxiety disorders and depression in different patients population as well as in the general population [14]. Scores 0–6 are defined as non-cases, 7–10 are defined as possible cases and score above 10 on each respective subscale are defined as cases.

### Burnout

Self-reported burnout was evaluated in this study using The Shirom-Melamed Burnout Questionnaire (SMBQ). The questionnaire includes 22 items measuring different aspects of burnout syndrome, including physical fatigue, emotional exhaustion, tension, listlessness and cognitive weariness as defined by Melamed and co-workers [15]. The SMBQ correlates strongly with the Maslach Burnout Inventory, another widely used instrument for measurement of burnout, and a mean score above 3.75 was used as a cut-off to define high burnout based on quartile splits [16]. Stenlund and co-workers reported the mean score of the total scale in patients with burnout to be 5.7 for females and 5.6 for males [17]. In this study, the median value of the SMBQ score for the total population (controls and patients) was used as cut-off to define high burnout. The median value was 4.3 in part one (N = 84) and 4.0 in part two (N = 177).

### Samples and Measurements

Fasting blood samples were collected in EDTA tubes during the patient's first visit to the clinic, before onset of treatment and all samples were taken before 09:30 AM. Healthy controls also visit the clinic in the morning for fasting blood sampling. Plasma was obtained by centrifugation of the blood samples at 4°C for 15 minutes at 1250 *g* and stored at −80°C before analysed.

#### Cytokines and growth factors

Plasma concentrations of epidermal growth factor (EGF), monocyte chemotactic protein-1 (MCP-1), vascular endothelial growth factor (VEGF), interleukin-1alpha (IL-1α), interleukin-1beta (IL-1β), interleukin-2 (IL-2), interleukin-4 (IL-4), interleukin-6 (IL-6), interleukin-8 (IL-8), interleukin-10 (IL-10), tumour necrosis factor-alpha (TNF-α) and interferone-gamma (IFN-γ) were assessed using an automated immunoassay system, Evidence, based on protein biochip array technology (Randox Laboratories Ltd, Crumlin, UK). The technique is by Fitzgerald *et al* [18]. All samples were analyzed in duplicates.

#### Comparing different analytic techniques to measure cytokines and growth factors

Optimal cut off values of MCP-1, EGF and VEGF for subjects at risk of being classified as ill were suggested by Asberg *et al* [11]. In order to use strictly defined cut-off values, similar results must be obtained even when different analytic measures are used. The initial analyses in this study were performed using a biochip immunoassay system from the same manufacturer (Randox Laboratories Ltd) as in the Asberg study [11]. The plasma levels of MCP-1 and VEGF were thus reanalyzed using the Meso Scale Delivery (MSD) Technology (Gaithersburg, Maryland). This was done eight months later, using the same samples, stored at −80°C until analysed and only individuals (both patients and controls) with valid measure from both analyses were included in the statistical analysis (n = 83 for MCP-1 and n = 65 for VEGF). EGF was not re-analysed as this protein was not included in the MSD platform.

#### CRP measurements

CRP levels were measured from serum by using turbidimetric analysis (Roche, Basel, Switzerland).

### Statistical Analysis

Statistical analyses of cytokines and CRP in plasma and serum were performed using Mann-Whitney unpaired non-parametric test. Differences in plasma levels of MCP-1 and VEGF obtained using RANDOX and MSD platforms were analyzed using Wilcoxon sign-ranked test. The concurrence between the two platforms was analysed using 95% limits of agreement. No data imputation was done and cytokines or growth factors with more than 50% non-detectable values were not considered meaningful to statistically analyse. Differences in depression and anxiety scores were assessed using chi-square test. For all tests, p<0.05 was considered statistically significant.

## Results

### Patients with Stress-Related Exhaustion Do Not Have Altered Cytokine Profile

The plasma levels of IL-1α, IL-1β, IL-2, IL-4, IL-6, IL-8, IL-10, IFNγ, TNFα, MCP-1, EGF and VEGF from female patients with ED and control subjects were analysed (n = 42+42). Due to the high number of non-detectable values, IL-1α (70% missing values), IL-4 (77% missing values), IL-10 (54% missing values) and IFNγ (70% missing values) where not considered meaningful to statistically analyse. No difference was seen between the controls compared to the patient group regarding the numbers of individuals with non-detectable values.

For IL-1β (24 controls and 25 patients), IL-2 (20 controls and 24 patients), IL-6 (33 controls and 28 patients), IL-8 (37 controls and 26 patients) and TNFα (42 controls and 40 patients) no statistical differences were seen between patients and controls (data not shown). For MCP-1, EGF and VEGF, no significant differences were observed between the two groups (Table 4). MCP-1, EGF and VEGF levels were further investigated in relation to the measures of self-reported depression, anxiety and burnout irrespective of the ED diagnose. Thus, both patients and control subjects were included in these analyses and divided into groups of high and low burnout according to the median split of SMBQ score as well as high and low anxiety and depression levels according the HAD scale recommended score for cases of anxiety disorder and depression (>10). No significant difference was seen between the groups for any of the analysis (Table 4). However, with p values of 0.083 for the anxiety score and 0.091 for depression, a trend can be seen that the high scoring cohorts actually tend to have lower MCP-1, which further contradicts the suggested biomarker function showed by Asberg and co-workers [11].

**Table 4:** Plasma levels (pg/ml) of MCP-1, VEGF and EGF in relation to Exhaustion disorder (ED) diagnosis, degree of burnout, anxiety and depression in female patients and healthy controls.

	Control	Patient	Burnout	Burnout	Anxiety	Anxiety	Depression	Depression
			<4.3	≥4.3	score ≤10	score >10	score ≤10	score >10
**MCP-1**	115±18.6	117±7.49	111±18.7	122±7.43	119±15.8	102±8.67	118±11.9	92.3±12.0
	(42)	(41)	(42)	(41)	(49)	(34)	(68)	(15)
		p = 0.771		p = 0.978		p = 0.083		p = 0.091
**EGF**	5.93±1.03	6.31±5.04	6.47±1.07	5.42±5.08	5.12±1.02	8.37±5.23	5.54±1.35	7.11±8.70
	(25)	(19)	(25)	(19)	(26)	(18)	(34)	(10)
		p = 0.696		p = 0.991		p = 0.272		p = 0.300
**VEGF**	8.98±6.20	11.6±1.54	10.6±5.99	10.4±1.62	9.23±5.26	10.7±1.76	11.3±3.73	9.03±1.99
	(33)	(37)	(34)	(36)	(39)	(31)	(56)	(14)
		p = 0.259		p = 0.747		p = 0.332		p = 0.445

Data are presented as median and standard error of the mean (SEM).

Due to the high number of non-detectable values, the number of observations varies and is listed within parenthesis for each measurement.

P values are given for respective analysis between the groups.

We also checked the impact of self-reported physical activity (4 graded scale), completed by 75 of the 84 subjects included in the study. No difference was seen between the different activity groups indicating that the level of physical activity does not contribute to the results. The use of nicotine did not influence the cytokine or growth factor levels either, nor did consumption of antidepressants.

**Figure 1:**
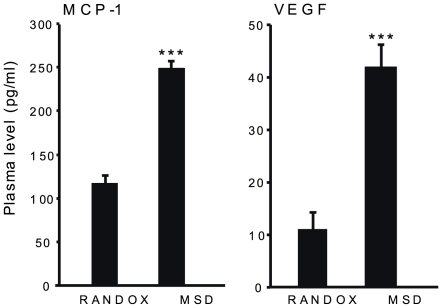
Plasma levels of MCP-1 and VEGF using different platforms. Plasma levels of MCP-1 (n = 83) and VEGF (N = 65) from patients and healthy controls measured using two different analysing methods. Using the MSD platform yielded significantly higher levels of both MCP-1 (2.1-fold) and VEGF (3.7-fold) compared to the RANDOX platform. Data are presented as median and SEM. *** p<0.001

Re-analysing the plasma levels of MCP-1 and VEGF from patients and controls by using the MSD platform revealed significantly higher median levels for both MCP-1 (N = 83) and for VEGF (N = 65) compared to the median level obtained by using the RANDOX platform (Figure 1). Similar to the results from the RANDOX platform, no significant differences were observed between patients and healthy controls (data not shown).

The discrepancy between the two platforms was analysed using 95% limits of agreement test [19]. When comparing the MCP-1 levels measured with the MSD platform with the levels obtained using the RANDOX platform, the mean difference was 131 pg/ml, with 95% limits of agreement ranging from −64 to 326 pg/ml. For VEGF, the mean difference was 34 pg/ml and the 95% limits of agreement ranged from −6 to 75 pg/ml.

### Patients with Stress-Related Exhaustion Have Increased Serum Levels of CRP

**Figure 2:**
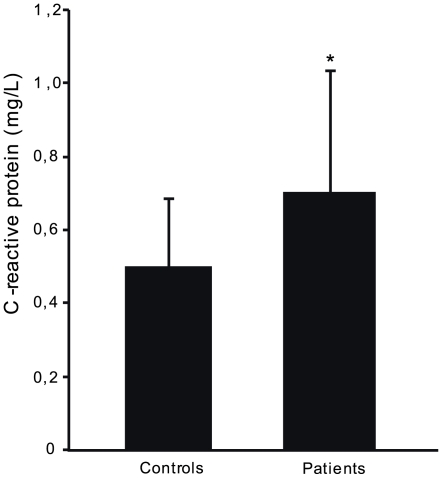
Serum levels of CRP. Serum levels of inflammatory marker CRP were significantly higher (1.4-fold) in patients with Exhaustion Disorder (n = 89) compared with control subjects (n = 88). Data presented as median and SEM. * p = 0.02

Although no differences in plasma levels of cytokines were observed between patients with exhaustion and control subjects, the degree of inflammation were further analyzed by measuring the inflammatory marker CRP in another group of patients fulfilling the diagnostic criteria for ED. The patients did have a significantly higher serum CRP concentration compared to healthy control subjects (Figure 2). The level of CRP was not significantly related to high depression, anxiety or burnout score (data not shown) and no difference was observed between patients using antidepressants and other patients (data not shown).

## Discussion

In this study, we measured the plasma levels of several cytokines and growth factors in patients with stress-related exhaustion and healthy controls. We found no statistically significant differences between the groups for any of the proteins measured. Thus, we were unable to replicate the findings by Asberg and co-workers suggesting that MCP-1, EGF and VEGF may be markers of long-term psychosocial stress [11]. Furthermore, using the measures of high burnout, depression or anxiety instead of exhaustion did not change this conclusion, since none of these measures were associated with the suggested markers of long-term stress.

There are several issues concerning the validity of cytokines and growth factors as stress markers. An important consideration is the large inter-individual variation in both ours and the previous study [11]. Furthermore, the circadian rhythm affects MCP-1 [20], VEGF [21] and EGF [22] levels and age is also known to affect circulating levels of several cytokines [23]. Elevated MCP-1 has been shown to be associated with depression [24], chronic pain diagnosis such as fibromyalgia [25] and chronic inflammatory diseases such as rheumatic arthritis [26]. In addition, plasma levels of MCP-1 are affected by a polymorphism in the MCP-1 gene [27].

Interestingly, we did not find an association between MCP-1 and depression as previously reported [28]. On the contrary we found a trend for a lower MCP-1 value with higher scores of both anxiety and depression. A majority of our patients fulfilled diagnostic criteria for depression but it is possible that these patients are somewhat different from other patient populations with depression since exhaustion is the most dominant clinical feature of the patients included in this study, and both depression and anxiety could be secondary to the exhaustion. This, however, remains to be thoroughly studied. Also, we noticed that it seems to be an discrepancy between the number of patients fulfilling the criteria for depression and those scoring above ten on self-reported scale for depression (HAD), but self-reported anxiety seems to be more congruent with the clinical diagnose. This need to be further studied in future work on these patients.

The Asberg study suggested that cut-off values of 243 pg/ml for MCP-1 and 7.80 pg/ml for VEGF could be used to predict the likelihood of being classified as ill due to long-term stress responses [11]. If this is to be generalized it is required that similar levels are obtained regardless of the analytic method used. We found that the average values of MCP-1 for the whole group were 117 and 249 pg/ml using the RANDOX and MSD platforms respectively, and for VEGF the average values were 11 and 42 pg/ml respectively. If the suggested cut-off values are applied on the MSD platform generated data in this study, all healthy controls would have been classified as ill according to their VEGF levels, and 64% according to their MCP-1 levels. The discrepancy in protein levels between the two platforms could be explained by several factors, such as the generation and purity of antibodies and reference proteins (different species or different techniques), the range of the standard curve, technical differences of the platforms and analysis sensitivity and specificity. Besides the difference in protein levels, the 95% limit of agreement between the two methods showed that the distribution of difference was very wide, indicating a high variability between the two different measurements which is rather remarkable since the same plasma samples were analysed. Given that these two analytic techniques show such a difference when samples from the same individuals are measured indicates that comparing absolute values between two different studies might not be possible. It seems more suitable to present data as a magnitude of change in relation to control levels and any general cut-off values for risk of illness might not be possible to use.

Although there were no differences in plasma levels of cytokines, we wanted to further investigate possible differences in inflammatory status between patients with exhaustion and controls. We found that the patients had small but significantly increased levels of CRP, which is in line with a previous study reporting correlation between CRP and degree of burnout in women [29], which could, however, not be confirmed in a smaller study [16]. CRP has also been associated with long-term stress [30] as well as with depression [31], [32] but data is not consistent [33]. The increased levels of CRP found in the patients in this study suggest that there could be a relation between long-term stress and low-grade inflammation and calls for more studies to confirm this. CRP has been shown to increase with age [34] but since the control subjects were a few years older, higher age does probably not account for this difference. There were no differences in gender or BMI between the groups and additional analysis using logistic regression confirmed that neither age, BMI nor gender contributed to the differences between the two groups.

There are several limitations that may affect the outcome of our study, one being the rather small number of patients included compared to the previous study by Asberg, as well as different characteristics of the populations studied [11]. This could partly explain the discrepancy of the results but if MCP-1, EGF and VEGF are to be used as stable markers of stress, they should be able to discriminate patients from controls even in smaller groups. Like in the previous study the measurements included was based on only one single blood sample. Several factors, such as nutrition, nicotine consumption, medication and physical activity should be controlled for when inflammation markers are studied. Information regarding most of these factors was available in this study and none were seen to affect the results.

In summary we conclude that MCP-1, EGF and VEGF may not be suitable as markers of prolonged psychosocial stress. However, low-grade inflammation seem to be present in patients suffering from exhaustion due to long-term psychosocial stress and it is plausible that some markers related to inflammation can be used as future stress markers. The large discrepancy between protein levels of suggested markers, measured with two different proteins assays, raises the question of using absolute values when comparing studies in which different analytic methods have been used.
